# Hydrogen peroxide as a central redox signaling molecule in physiological oxidative stress: Oxidative eustress^☆^

**DOI:** 10.1016/j.redox.2016.12.035

**Published:** 2017-01-05

**Authors:** Helmut Sies

**Affiliations:** a Institute of Biochemistry and Molecular Biology I, Heinrich Heine University Düsseldorf, Düsseldorf, Germany; b Leibniz Institute for Research in Environmental Medicine, Heinrich Heine University Düsseldorf, Düsseldorf, Germany

**Keywords:** Oxidative stress, NADPH oxidases, Mitochondria, Peroxiporins, Redox regulation, H_2_O_2_

## Abstract

Hydrogen peroxide emerged as major redox metabolite operative in redox sensing, signaling and redox regulation. Generation, transport and capture of H_2_O_2_ in biological settings as well as their biological consequences can now be addressed. The present overview focuses on recent progress on metabolic sources and sinks of H_2_O_2_ and on the role of H_2_O_2_ in redox signaling under physiological conditions (1–10 nM), denoted as oxidative eustress. Higher concentrations lead to adaptive stress responses *via* master switches such as Nrf2/Keap1 or NF-κB. Supraphysiological concentrations of H_2_O_2_ (>100 nM) lead to damage of biomolecules, denoted as oxidative distress. Three questions are addressed: How can H_2_O_2_ be assayed in the biological setting? What are the metabolic sources and sinks of H_2_O_2_? What is the role of H_2_O_2_ in redox signaling and oxidative stress?

## Highlights

• H_2_O_2_ is operative in redox sensing and redox signaling.
• H_2_O_2_ reacts with metal centers and with sulfur/selenium compounds.
• H_2_O_2_ links redox biology to phosphorylation/dephosphorylation.
• Physiological (low-level, nM) steady-state of H_2_O_2_ is maintained in oxidative eustress.
• Supraphysiological (pathological) level of H_2_O_2_ leads to oxidative distress.

## Graphical abstract

**Figure:**
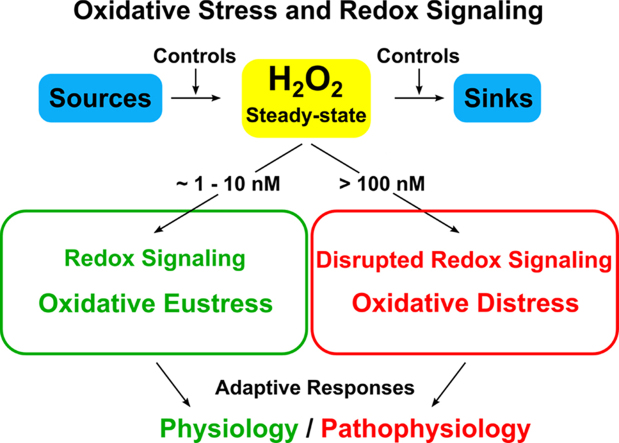

## 1 Introduction

Surprisingly (or unsurprisingly, in hindsight) hydrogen peroxide emerged as the major redox metabolite operative in redox sensing, signaling and redox regulation (see [1] for recent review)·H_2_O_2_ is recognized as being in the forefront of transcription-independent signal molecules, in one line with Ca^2+^ and ATP [2], [3]. As a messenger molecule, H_2_O_2_ diffuses through cells and tissues to initiate immediate cellular effects, such as cell shape changes, initiation of proliferation and recruitment of immune cells. It became clear that H_2_O_2_ serves fundamental regulatory functions in metabolism beyond the role as damage signal [4]. The metabolic and regulatory role of this oxygen metabolite has been increasingly recognized [5]. It serves as a key molecule in the Third Principle of the Redox Code, which is: “Redox sensing through activation/deactivation cycles of H_2_O_2_ production linked to the NAD and NADP systems to support spatiotemporal organisation of key processes” [6]·H_2_O_2_ occurs in normal metabolism in mammalian cells [7] and is a key metabolite in oxidative stress (see [8]). The term “oxidative eustress” [9], [10], which denotes physiological oxidative stress, may serve in the distinction from excessive load, “oxidative distress”, causing oxidative damage (see also [11], [12], [13], [14]). The general concept of eustress *vs*. distress was formulated in Refs. [15], [16].

## 2 Timeline: hydrogen peroxide in chemistry and biology

**Fig. 1:**
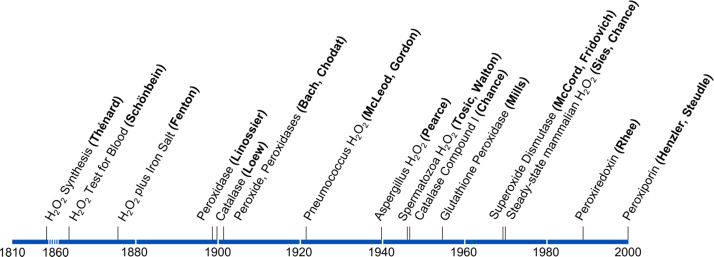
Timeline of hydrogen peroxide in chemistry and biology.

Shortly after the discovery of oxygen by Lavoisier, Scheele and Priestley in the late 18th century it was Thénard who, in 1818, first synthesized H_2_O_2_ and found that blood disintegrates it. The role of H_2_O_2_ in cellular respiration was a matter of intense debate between Otto Warburg and Heinrich Wieland in the 1920's. The early detection of H_2_O_2_ efflux from pneumococcus or from aspergillus focused on cell-killing properties. It took until 1970 that the physiological occurrence of H_2_O_2_ as a normal attribute of mammalian metabolism was demonstrated. The timeline (Fig. 1) also shows the appearance, on the horizon of research, of the Fenton reaction and the naming of peroxidases and catalase in the 19th century. Glutathione peroxidase (Mills), superoxide dismutase (McCord and Fridovich) and peroxiredoxins (Rhee) were further milestones. The transport of H_2_O_2_ across membranes by water channels (Henzler and Steudle), by specific aquaporins designated as peroxiporins, concluded that development in the 20th century.

## 3 How can H_2_O_2_ be assayed in the biological setting?

### 3.1 Steady-state of catalase Compound I

Hydrogen peroxide is now well-known as a normal metabolite of oxygen in aerobic metabolism of cells and tissues. As mentioned, this was first shown in 1970 by the detection, in the near-infrared, of catalase Compound I in the intact perfused liver [7]. Catalase Compound I is the product of the reaction of H_2_O_2_ with catalase hame, denoted here in a simplified manner (Reaction (1)) [17], [18]. It can decompose upon reaction with a second H_2_O_2_ molecule in the catalatic reaction, releasing oxygen (Reaction (2)), or it can decompose in the peroxidatic reaction with a hydrogen donor AH_2_, *e.g*. methanol (Reaction 3):

(1) $$\begin{array}{c} \mathrm{Formation}\;\mathrm{of}\;\mathrm{Compound}\;I:\;{\text{catalase}-\text{Fe}}^{3+}+H_{2}O_{2}\to {\text{catalase}-\text{FeO}}^{3+}+H_{2}O\\ \;\;\;\;\;\;\;\;\;\;\;\;\;\;\;\;\;\;\;\;\;“Compound\;I” \end{array}$$

(2) $$\text{Catalatic}\;\text{decomposition}:\;\;{\text{catalase}-\text{FeO}}^{3+}{+H}_{2}O_{2}\to {\text{catalase}-\text{Fe}}^{3+}{+\;\text{O}}_{2}{+\;\text{H}}_{2}O\;\mathrm{In}\;\text{sum}:\;2\;H_{2}O_{2}\to O_{2}+2\;H_{2}O$$

(3) $$\begin{array}{c} \mathrm{Peroxidatic}\;\mathrm{decomposition}:\;{\text{catalase}-\text{FeO}}^{3+}+{\mathrm{AH}}_{2}\to {\text{catalase}-\text{Fe}}^{3+}+A+H_{2}O\\ \;\;\;\;\;\;\;\;\;\;\;\;\;\;\;\;“hydrogen\;donor” \end{array}$$

$\mathrm{In}\;\text{sum}:\;H_{2}O_{2}{+\text{AH}}_{2}\to A+2\;H_{2}O$

The charge-transfer band of catalase Compound I at 660 nm [17] is useful for spectrophotometry of cells or organs because of low light-scattering and practically no optical interference by other pigments in the near-infrared region of the spectrum. Unequivocal proof of the existence of H_2_O_2_ in normally respiring liver cells was provided by noninvasive organ spectroscopy: Catalase Compound I was shown to decrease rapidly when the oxygen concentration was lowered and, importantly, when a hydrogen donor (*e.g*. methanol) for the peroxidatic reaction (Reaction (iii)) of catalase was infused [7]. Using stepwise ‘titration’ with low methanol concentrations, the rate of cellular H_2_O_2_ production was determined to be 50 nmol H_2_O_2_ per gram of liver wet weight per min, which corresponds to 2–3% of total oxygen uptake [19]. That rate was increased in several metabolic conditions (Table 1) [19]. The method has also been used with isolated hepatocytes, also shown in Table 1 [20]. The steady-state concentration of H_2_O_2_ in the intact cell, overall, was calculated to be of the order of 10 nM [19], [21]. Early methods to quantify H_2_O_2_ formation included assays of the peroxidatic reaction, e.g*.* the use of [^14^C]-methanol, and others [22], [23].

**Fig. 2:**
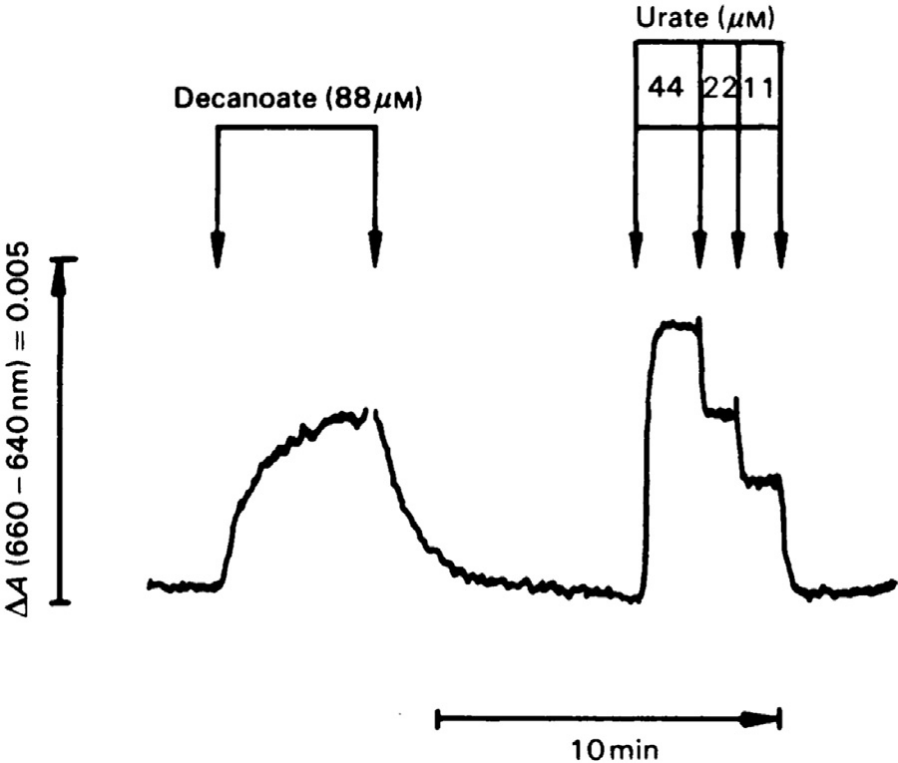
Quantification of H_2_O_2_ production in intact perfused rat liver during decanoate oxidation. Catalase Compound I is monitored at 660–640 nm continuously against time by organ spectrophotometry. Calibration of the decanoate response is performed against the urate response. With the 1:1 stoichiometry of urate:H_2_O_2_ and measurement of the rate of urate removal in the effluent perfusate, the decanoate response is quantified to indicate an extra H_2_O_2_ production of 80 nmol H_2_O_2_/min per gram liver wet weight in this experiment. For details, see [22], [23].

Another method of quantification of extra H_2_O_2_ production in intact cells is illustrated in Fig. 2. Upon infusion of decanoate as a fatty-acid substrate for H_2_O_2_ production, there is an increase in the catalase Compound I signal at 660–640 nm, which can be calibrated against that obtained with infusion of urate, a substrate producing known amounts of H_2_O_2_ by urate oxidase [22], [23]. In isolated hepatocytes, a comparison of endogenous H_2_O_2_ production rates with the catalatic evolution of O_2_ from infused H_2_O_2_ provided another means to analyse Compound I steady-states [24].

### 3.2 Genetically encoded fluorescent protein indicators of H_2_O_2_

The development of the HyPer probe as genetically encoded fluorescent indicator for H_2_O_2_ in intact cells and tissues was a landmark [25]. This new type of probe provided insight into spatiotemporal organisation [26]. Thus, it became possible to examine the dynamics of H_2_O_2_ in subcellular compartments [27]. HyPer is also sensitive to pH. Dissection of pH responses from responses in H_2_O_2_ is possible with the SypHer [28] and SypHer2 probes [29].

Probes such as roGFP2-Orp1 provide another type of tool for assaying H_2_O_2_, exhibiting kinetics of oxidation and reduction similar to HyPer [30], [31]. Real-time monitoring of basal H_2_O_2_ levels has been used with peroxiredoxin-based probes [32].

### 3.3 Exomarkers,’nonredox’ exogenous probes;small molecule fluorescent markers

A variety of other approaches for H_2_O_2_ detection is available, not to be discussed here in detail (e.g. [33], [34], [35]).

## 4 What are the metabolic sources and sinks of H_2_O_2_?

### 4.1 Sources: NAD(P)H oxidases, mitochondrial respiratory chain, diverse oxidases

**Fig. 3:**
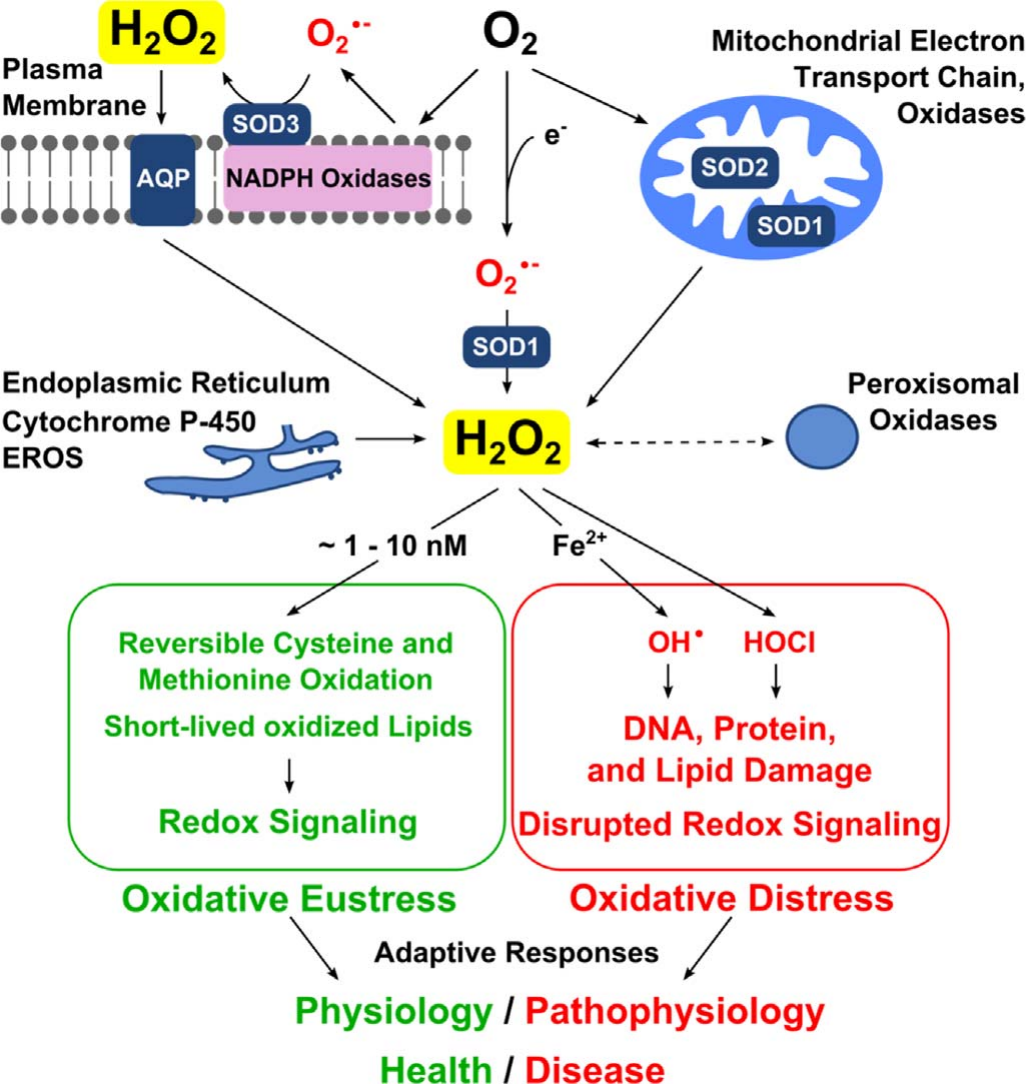
Role of hydrogen peroxide in oxidative stress. *Top*: Endogenous H_2_O_2_ sources include NADPH oxidases and other oxidases (membrane-bound or free) as well as the mitochondria. The superoxide anion radical is converted to hydrogen peroxide by the three superoxide dismutases (SODs 1,2,3). Hydrogen peroxide diffusion across membranes occurs by some aquaporins (AQP), known as peroxiporins. *Bottom*: In green, redox signaling comprises oxidative eustress (physiological oxidative stress). In red, excessive oxidative stress leads to oxidative damage of biomolecules and disrupted redox signaling, oxidative distress. (For interpretation of the references to color in this figure legend, the reader is referred to the web version of this article.)

Numerous one- or two-electron reduction reactions have been identified as sources of H_2_O_2_ **(**Fig. 3**)**. The major enzymatic generators are the NADPH oxidases (Nox) [36], [37] and the mitochondrial respiratory chain [38], [39], [40], as well as a considerable number of oxidases. A total of 31 human cellular hydrogen peroxide generating enzymes has been compiled [41]. The one-electron reduction sources are functionally tightly linked to the superoxide dismutases, SOD1, SOD2, SOD3, with their cytosolic, mitochondrial matrix and extracellular locations, respectively. Other cell compartments also contribute to H_2_O_2_ production, *e.g*. the endoplasmic reticulum and the peroxisomes.

### 4.2 Sinks: catalases, peroxidases, peroxiredoxins

While the catalatic reaction of catalase (Reactions (i) and (ii)) is a dismutation reaction, regenerating one molecule of oxygen, the other sinks of H_2_O_2_ lead to water by reduction. Certain cysteinyl residues in peroxiredoxins or selenocysteinyl residues in glutathione peroxidases are highly reactive, the second-order rate constants for the reaction with H_2_O_2_ being of the order of 10^7^ M^-1^s^-1^ (see [42]). A number of important peroxidases utilize H_2_O_2_ as substrate, *e.g*. myeloperoxidase, eosinophil peroxidase, lactoperoxidase.

The relative contributions of catalase and glutathione peroxidase in H_2_O_2_ removal depend on the site of generation and the enzymatic equipment [43], [44], [45], [46]. Catalase is a predominantly peroxisomal enzyme. Therefore, it is either a localized sink for H_2_O_2_ produced by peroxisomal oxidases, or peroxisomes can be a sink at the cell-scale level (see comment further below). An overall assay for the rate of removal of extracellular hydrogen peroxide has been developed for cell culture experiments [47].

### 4.3 H_2_O_2_ Compartmentation: role of aquaporins as peroxiporins

Aquaporins (AQP) facilitate H_2_O_2_ to cross membranes [48]. Specific AQPs play a functional role in H_2_O_2_ translocation, for which they are called peroxiporins [49], [50].

### 4.4 Spatiotemporal control, H_2_O_2_ nanodomains

**Fig. 4:**
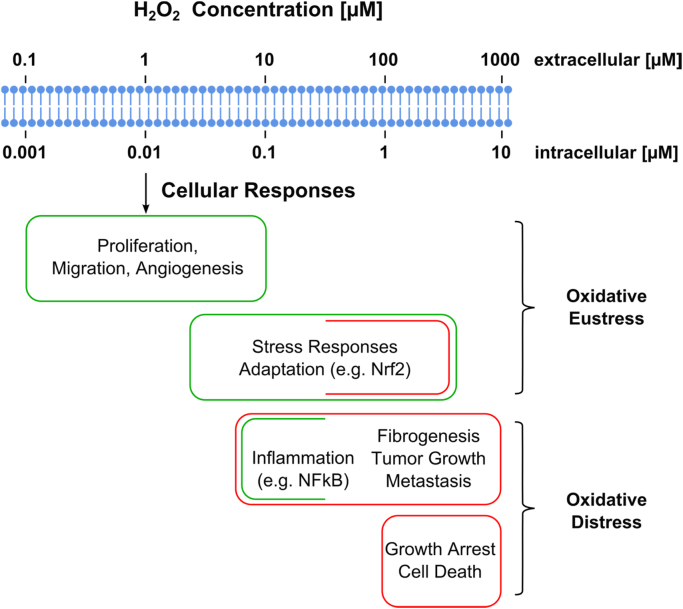
Estimated ranges of hydrogen peroxide concentration in oxidative stress with regard to cellular responses. The intracellular physiological range likely spans between 1 and 10 up to approx. 100 nM H_2_O_2_; the arrow indicates data from normally metabolizing liver. Stress and adaptive stress responses occur at higher concentrations. Even higher exposure leads to inflammatory response, growth arrest and cell death by various mechanisms. Green and red coloring denotes predominantly beneficial or deleterious responses, respectively. An estimated 100-fold concentration gradient from extracellular to intracellular is given for rough orientation; this gradient will vary with cell type, location inside cells and the activity of enzymatic sinks (see text). (For interpretation of the references to color in this figure legend, the reader is referred to the web version of this article.)

Spatial distribution of H_2_O_2_ in cells and tissues is not uniform. There are substantial gradients, both from extracellular to intercellular and between subcellular spaces [51], [52], [53]. It appears from a search of the available literature that H_2_O_2_ concentration in blood plasma is about 1–5 µM [54], which would be more than 100-fold higher than that estimated to occur within cells (see Fig. 4). Interestingly, even within subcellular organelles, there are H_2_O_2_ gradients·H_2_O_2_ in the mitochondrial cristae space originates largely from mitochondrial Complex III, whereas mitochondrial Complexes I and II contribute to mitochondrial matrix H_2_O_2_ [55]. In the cristae subspace “redox nanodomains” have been described, which are induced by and control calcium signaling at the ER-mitochondrial interface [56]·H_2_O_2_ transients sensitize calcium ion release to maintain calcium oscillations [56].

There are also circadian redox oscillations, and these are likely to comprise oscillations in H_2_O_2_ concentration. Peroxiredoxins exhibit pronounced circadian rhythmicity [57], [58]. There is reciprocal control of the circadian clock and the cellular redox state (see [59]).

The thioredoxin reductase-1/thioredoxin [60] and glutathione reductase/glutathione systems [61] appear to control the actions of H_2_O_2_ on master switches such as Nrf2/Keap1. Nuclear and cytosolic peroxiredoxin-1 differentially regulate NF-κB activities, indicating that the balance in subcellular H_2_O_2_ metabolism provides specificity in redox signaling [62].

For orientation on H_2_O_2_ concentration ranges, Fig. 4 presents physiological ranges, spanning from normal processes to adaptive ones (stress responses), denoted as “oxidative eustress”. Higher concentrations evoking inflammatory responses and others, ultimately leading to growth arrest and cell death, are denoted as “oxidative distress”. It may be mentioned that it is difficult to conceptually discern between these ranges, because inflammation, for example, certainly includes elements positive for the organism, such as phagocyte activity. So does autophagy [63], [64], mitophagy [65] and cell death in the forms of apoptosis, ferroptosis and necroptosis [66]. Assignment of a given H_2_O_2_ concentration to the category of eustress or of distress may vary with cell type, with the level of complexity (comparison of isolated cell/tissue and the whole organism), or with the duration of the exposure to H_2_O_2_. Considering the level of the whole organism, mucosal barrier tissues participate in immune defense against infection. The release of nano- to submicromolar H_2_O_2_ was shown to disrupt the tyrosine phosphorylation network in several bacterial pathogens as a host-initiated antivirulence strategy [67].

Estimations of kinetic parameters of H_2_O_2_ interactions have been performed, and models were established to improve our understanding of the complex processes in redox signaling [68], [69], [70], [71].

In Fig. 4, the gradient between extracellular and intracellular H_2_O_2_ concentrations is indicated, as rough orientation, to be about 100-fold, which is 10-fold higher than previous estimations [5]. Recent calculations based on results obtained with genetically encoded H_2_O_2_ probes came to even higher values, 200- to 500-fold [72] or 650-fold [52]; these rely on the information from the intracellular subspace where the detector probe is located. It is conceivable, of course, that there are spaces with even zero H_2_O_2_ concentration, which would make the gradient tend toward infinity.

## 5 What is the role of H_2_O_2_ in redox signaling and oxidative stress?

### 5.1 Mechanism

Because of its physicochemical properties, H_2_O_2_ is capable of serving as messenger to carry a redox signal from the site of its generation to a target site. Among the various oxygen metabolites, H_2_O_2_ is considered most suitable for redox signaling [1], [73]. Redox regulation can take place via control of single enzymatic activity or at the transcriptional level. Hydrogen peroxide modulates the activity of transcription factors: in bacteria (OxyR and PerR) (see [74]), in lower eukaryotes (Yap1, Maf1, Hsf1 and Msn2/4) and in mammalian cells (AP-1, NRF2, CREB, HSF1, HIF-1, TP53, NF-κB, NOTCH, SP1 and SCREB-1) (see [1]).

The major impact on redox regulation occurs via the thiol peroxidases (see *Sinks* above) [75], which involves, to a large degree, reversible protein cysteine oxidation to the sulfenylated form (Fig. 3) [76], [77], [78]. According to the Second Principle of the Redox Code, “the redox proteome is organized through kinetically controlled sulfur switches linked to NAD and NADP systems” [6], and H_2_O_2_ plays a central role in it as an oxidant.

The linkage of redox reactions to protein phosphorylation/dephosphorylation is given by the redox sensitivity of proteine tyrosine phosphatases (PTP). Thus, oxidation and thereby inactivation of PTPs would increase steady-state protein phosphorylation. Examples are PTEN (phosphatase and tensin homolog), Cdc25 phosphatases, and PTP1B (protein tyrosine phosphatase 1B) (see [79]).

Protein phosphatase 1 (PP1) is redox-inhibited by oxidation of its metal center [80]. The intracellular labile iron pool is a determinant of H_2_O_2_-induced redox signaling [81], [82].

### 5.2 Targets

It follows that specificity and fine-tuning is exerted through control of sources and sinks. Regarding sources, the fine-control of NADPH oxidases by physical and chemical cues is pivotal (see [83]). Mitochondrial control by redox switches coordinates protein activity, localization and stability (see [84], [85]). The cysteine redox proteome has been studied thoroughly [41], [86], [87]. The median percentage oxidation of cysteine residues in the proteome is between 5% and 12%, and this can be increased to >40% by adding oxidants [86]. Regarding sinks, much interest has focused on the peroxiredoxins [88]. For example, peroxiredoxin-2 acts as highly sensitive primary H_2_O_2_ receptor which specifically transmits oxidative equivalents to the redox-regulated transcription factor STAT3, forming a redox relay for H_2_O_2_ redox signaling [89].

### 5.3 Processes

As many fundamental biological processes have a component of redox control, exhaustive coverage is beyond the scope of the present article. Major such processes are hypoxia, inflammation (“inflammasome“), autophagy, apoptosis, wound healing, proliferation, muscle contraction, circadian rhythm, stem cell self-renewal, tumorigenesis and aging (see above and Refs. [8], [90]).

Here, just a few recent aspects may be mentioned which illustrate the growing recognition of H_2_O_2_ in direct involvement: Laminar shear-stress mediates H_2_O_2_ formation in bovine aortic endothelial cells [91]. NADPH oxidase 4 (Nox4) has a protective vascular function, its deletion causes apoptosis [92]. Nox4 contributes considerably, by about one-third, to cellular H_2_O_2_ formation in vascular endothelium [92]. The redox inhibition of protein phosphatase 1, mentioned above, by Nox4 regulates elF2-alpha-mediated stress signaling [80]. Specialized cell-types such as vascular smooth muscle cells are good examples of the complex nature of the role of H_2_O_2_ in pathophysiology [93].

In injured zebrafish larvae, a newly generated H_2_O_2_ gradient mediates rapid leukocyte recruitment [94]. Cell proliferation subsequent to amputation of the tail of tadpoles involves increases in H_2_O_2_ concentration, as indicated by the HyPer probe (assuming that pH effects do not contribute significantly to the signal) [95]. The transients in H_2_O_2_ are embedded in concerted action with other signals in the process of wound responses mentioned in the Introduction above [96]·H_2_O_2_ controls axon pathfinding of retinal ganglion cells projecting towards the tectum in zebrafish [97].

The control of H_2_O_2_ gradients by peroxiporins (see above) allows for further possibilities of fine-tuning with impact on cell signaling and survival, as shown for AQP8 [98]. AQP3 is required for Nox-derived H_2_O_2_ signaling upon growth factor stimulation [99]. AQP3 was also shown to mediate H_2_O_2_ uptake to regulate downstream signaling in tumor necrosis factor-dependent activation of NF-κB [100]. The endoplasmic reticulum membrane is recognized as another important site of modulation of H_2_O_2_ traffic by aquaporins, notably by AQP11 [101].

## 6 Outlook

While encouraging progress has been made in understanding the biological significance of low levels of H_2_O_2_ in cells and tissues, much is yet to be discovered. New tools with improved sensitivity, specificity and selectivity promise better insight into spatiotemporal organisation by dynamic imaging [102], [103].

### 6.1 Quantification and imaging: the “H_2_O_2_ landscape”

The detailed description of the spatial and temporal pattern of H_2_O_2_ concentrations in cells and tissues is a major challenge. Overall intracellular H_2_O_2_ production rates as given in Table 1 can serve as a first approximation. Clearly, substantial subcellular gradients exist, from practically zero H_2_O_2_ concentration to localized high concentrations *e.g.* in vesicles. The use of catalase Compound I for assaying H_2_O_2_ steady-states rests on a number of conditions, not elaborated here in detail; it could be argued that there is overestimation or, conversely, underestimation of total cellular rates and subcellular concentrations of H_2_O_2_, depending *e.g.* on the activity of peroxiredoxins. While it is uncontested that catalase is located in the peroxisomal matrix, other locations of catalase activity are also relevant, such as the mitochondria [104] and even the extracellular space [105]. Using D-amino acid oxidase as genetically encoded producer of H_2_O_2_, in conjunction with the fused HyPer probe, it becomes possible to examine intracellular H_2_O_2_ dynamics quantitatively [106]·H_2_O_2_ microdomains in receptor tyrosine kinase signaling are now amenable to imaging [107].

Mitochondrial dynamics in terms of fusion and fission is subjected to short-term regulation by redox signaling [108]. Conversely, the assembly of mitochondrial Complex I into supercomplexes determines, for example, the differential production of reactive oxygen species in neurons and astrocytes [109]. Addressing specific sites of mitochondrial H_2_O_2_ production may have therapeutic potential [110].

### 6.2 Broader context: relation to Ca^2+^

The distinction of oxidative eustress and oxidative distress may occur at a fine borderline, embedded in other fundamental control systems regulated by ion signals, notably calcium ions [111], [112], [113]. The H_2_O_2_ nanodomains mentioned above [56] support the emerging concept that Ca^2+^ signaling and the luminal redox state of the endoplasmic reticulum are intertwined, especially at the mitochondria-associated membranes (MAM) [114], [115]. Associated pH transients are accessible as well [29].

Modulation and propagation of H_2_O_2_ signals is also subject to intercellular communication. The microenvironment surrounding particular cells, notably the extracellular matrix (ECM), modifies the signal [116]. Gap junctional communication via connexin channels between cells may lead to “local oxidative stress expansion” [117].

### 6.3 Note

This article focused on H_2_O_2_ as a central redox signaling molecule. Other reactive oxygen species and reactive nitrogen species, not treated here, also have a role in redox regulation. For example, the superoxide anion radical reacts with FeS-centers, *e.g*. in aconitase, and by its reaction with nitric oxide peroxynitrite is formed which, in turn, modifies proteins by tyrosine nitration. S-Glutathionylation processes as well as reactions of hydrogen sulfide and its congeners are also important. This is illustrated by the secretion of S-glutathionylated proteins under oxidative stress [118].

### 6.4 Upshot

The concept of constitutive cellular low-level H_2_O_2_ steady-states is gaining increasing recognition. The localized function in modulating and maintaining key target functions by redox reactions is the essence of physiological oxidative stress, called *oxidative eustress*. Localized high levels of H_2_O_2_ occur in *oxidative distress*, exemplified by cell-killing in the respiratory burst of neutrophils [119] or in toxicity studies [120] involving H_2_O_2_ generation. A challenge for refined analysis will be the spatiotemporal and functional dissection of these two extremes in cells and tissues.

## Figures and Tables

**Table 1:** H_2_O_2_ production rate in intact liver or in isolated hepatocytes. Intact hemoglobin-free perfused liver [19] or isolated hepatocyte [20] data were obtained by methanol titration of catalase Compound I. For discussion, see [4], [21].

**Substrate or inhibitor**	**H**_**2**_**O**_**2**_**production rate**	
	(nmol H_2_O_2_/min)	
	(per g of liver wet wt) (per 10^6^ hepatocytes)	
	Ref. [19]	Ref. [20]
Control	49	1.5
+ octanoate	170	4.0
+ glycolate	490	13.9
+ antimycin	75	
